# Effect of Regular Yoga Practice on Respiratory Regulation and Exercise Performance

**DOI:** 10.1371/journal.pone.0153159

**Published:** 2016-04-07

**Authors:** Eveline Beutler, Fernando G. Beltrami, Urs Boutellier, Christina M. Spengler

**Affiliations:** 1 Exercise Physiology Lab, Institute of Human Movement Sciences, ETH Zurich, Winterthurerstrasse 190, 8057, Zurich, Switzerland; 2 Zurich Center for Integrative Human Physiology (ZIHP), University of Zurich, Winterthurerstrasse 190, 8057, Zurich, Switzerland; Fondazione G. Monasterio, ITALY

## Abstract

Yoga alters spontaneous respiratory regulation and reduces hypoxic and hypercapnic ventilatory responses. Since a lower ventilatory response is associated with an improved endurance capacity during whole-body exercise, we tested whether yogic subjects (YOGA) show an increased endurance capacity compared to matched non-yogic individuals (CON) with similar physical activity levels. Resting ventilation, the ventilatory response to hypercapnia, passive leg movement and exercise, as well as endurance performance were assessed. YOGA (n = 9), compared to CONTROL (n = 6), had a higher tidal volume at rest (0.7±0.2 vs. 0.5±0.1 l, p = 0.034) and a reduced ventilatory response to hypercapnia (33±15 vs. 47±15 l·min^-1^, p = 0.048). A YOGA subgroup (n = 6) with maximal performance similar to CONTROL showed a blunted ventilatory response to passive cycling (11±2 vs. 14±2 l·min^-1^, p = 0.039) and a tendency towards lower exercise ventilation (33±2 vs. 36±3 l·min^-1^, p = 0.094) while cycling endurance (YOGA: 17.3±3.3; CON: 19.6±8.5 min, p = 0.276) did not differ. Thus, yoga practice was not associated with improved exercise capacity nor with significant changes in exercise ventilation despite a significantly different respiratory regulation at rest and in response to hypercapnia and passive leg movement.

## Introduction

The scientific interest in yoga has increased substantially in recent years and a number of physiological effects of yoga, including physical postures (asanas; [1, 2]), controlled breathing (pranayama; [3, 4–6]) and meditation [7], have been explored.

Focusing on the respiratory aspect of yoga, Pranayama generally involves controlled breathing techniques affecting the respiratory rhythm, namely through prolongation and shortening of breaths, and sometimes breath-holding, all implying voluntary control of respiratory muscles. These voluntary acts influence the breathing pattern, which is normally determined by the autonomic respiratory control center in the brain. There is evidence that voluntary control of breathing (practiced for 6–10 weeks) induces persistent alteration of the breathing pattern at rest, shown in a reduced breathing frequency (f_R_) [2, 3, 8–10]. On the other hand, some degree of controversy exists concerning changes in lung function, as improvements following yoga training are often [2, 8–12] but not always [3, 13, 14] reported.

Additionally, Spicuzza et al., [15] and Stanescu et al., [13] found that long-term yoga practice reduced chemoreflex sensitivity to hypoxia and hypercapnia, while blunted hypoxic ventilatory response (HVR) has also been shown both while breathing a modified gas mixture and at high altitude (hypobaric hypoxic conditions) in subjects with a broad experience in yoga practice [16]. Interestingly, a similarly reduced HVR and hypercapnic ventilatory response (HCVR) was described in athletes compared to sedentary controls [17] and in untrained subjects who have completed a 5-week period of cycling endurance training [18]. Therefore, a connection between changes in chemoreceptor sensitivity induced by practicing breathing exercises and physical performance seems plausible. Since the magnitude of the ventilatory response during exercise influences endurance capacity [19], this mechanism could be postulated for subjects practicing yoga, suggesting a lower ventilation during exercise, ultimately resulting in larger endurance capacity.

In fact, several studies indicate that regular yoga training could influence exercise performance in healthy subjects, shown in increased maximal tolerable workload (W_max_) and decreased oxygen consumption during equal workload [20–23] or a higher maximal oxygen consumption (V̇O_2peak_) after varying periods of yoga training [1, 14, 24, 25]. These results raise the question of whether the improvements in exercise performance are associated with an altered ventilatory response during exercise in subjects practicing yoga.

Therefore, the present study combined different assessments to determine whether long-term yoga practice is associated with (i) a modified breathing pattern at rest, (ii) a reduced ventilatory response to CO_2_, (iii) an extended breath-hold duration, (iv) a reduced ventilatory response to passive leg movement and (v) to exercise, and finally (vi) whether these changes are associated with increased endurance capacity. For this purpose, ventilation and breathing pattern at rest, lung function, respiratory muscle strength, breath-hold duration, HCVR (to investigate chemoreceptor sensitivity), the ventilatory response to passive leg movement and to exercise, in addition to exercise endurance capacity, were assessed.

## Methods

### Subjects

Fifteen healthy female subjects were enrolled in the study, either in the YOGA group (n = 9) or in the age-, height-, weight- and fitness level-matched control (CON) group (n = 6). Physical activity levels was matched based on modality, duration and intensity, excluding the time spent practicing yoga in the YOGA group. The YOGA group participants were all yoga instructors, practicing yoga for an average of 7.1 ± 3.5 h·wk^-1^, including pranayama for 3.0 ± 1.6 h·wk^-1^ for the past 8.1 ± 6.6 years. All participants were between 18–45 years of age, had a body mass index (BMI) of 18–25 kg·m^-2^, normal lung function, did not smoke or take any medications and were recreationally physically active. None of the participants suffered from acute or chronic diseases (assessed by questionnaire), had acute injuries, nor did participants take part in any activities that could possibly influence respiration such as singing or playing a wind instrument on a regular basis (i.e. ≥ 30 min·wk^-1^). For CON subjects, additional exclusion criteria were performing yoga or any kind of respiratory exercise or respiratory training.

After detailed oral and written information regarding all procedures and possible risks, each subject signed a written consent prior to participation. The study was approved by the ethics committee of ETH Zurich and was performed according to the Declaration of Helsinki (2008 Revision).

Subjects were advised to keep their physical activity level constant throughout the entire testing period and were instructed to arrive at the laboratory in a rested state, with a minimum of 7 h sleep during two preceding nights of each testing day. In addition all subjects had to avoid strenuous exercise 48 h prior to each testing session and avoid any exercise the day before and on test days. Each subject was also asked to consume a high-carbohydrate meal for dinner the day before test days and to refrain from caffeine and alcohol intake on test days prior to testing.

### Study protocol

Subjects attended the laboratory on 3 different occasions, separated by at least 48 h. Since the effect of the menstrual cycle on exercise performance is still debated in the current literature [26] and effects of progesterone on the pulmonary system, including hyperventilation and an increase in resting chemosensitivity to CO_2_ and O_2_, have been established [27], all measurements were conducted between the 7^th^ and 14^th^ day after ovulation, the longest period with little change in hormone concentrations.

All measurements were performed at the same time of day within each subject, but were spread over the day between subjects to distribute assessments over the potentially influencing endogenous circadian rhythm [28].

On the first day, lung function and respiratory muscle strength were measured for familiarization. On the second day, subjects performed lung function and respiratory muscle strength measurements again. After a 15-min resting period, subjects performed an incremental cycling test (ICT) to exhaustion. On the third day, subjects performed breath-hold testing followed, after a 10-min break, by 2 HCVR-tests that were separated by a 15-min break. Finally, after a 30 min break, the test including passive leg movement and a subsequent all-out constant load cycling endurance test (CET) was conducted. Tests are described in detail below.

### Lung function and respiratory muscle strength

An ergospirometric device (Oxycon Pro, Jaeger, Höchberg, Germany) was used to assess lung function with precise volume measurements by means of a lightweight bi-directional digital volume transducer, attached directly to the mouthpiece and calibrated before each test, using a 3L syringe (Hans Rudolph, Kansas City, MO, USA).

Forced vital capacity (FVC), forced expired volume in one second (FEV_1_), forced inspiratory volume in one second (FIV_1_), peak expiratory flow rate (PEF), peak inspiratory flow rate (PIF) and maximal voluntary ventilation (MVV) were measured adhering to ATS/ERS standard procedures [29].

Respiratory muscle strength was determined using a hand-held mouth pressure meter, equipped with piezo resistive pressure sensing technology (Micro Medical, Kent, UK). Maneuvers were performed according to the ATS/ERS statement [30] on respiratory muscle testing. Maximal inspiratory mouth pressure (MIP) measurements were initiated at residual volume (RV), maximal expiratory mouth pressure (MEP) measurements at total lung capacity (TLC) using a flanged mouthpiece [31], with the subject seated in an upright position. Sniff nasal inspiratory pressure (SNIP) was measured by means of a plug occluding one nostril, initiated at functional residual capacity (FRC). A minimum of 5 well-executed measurements was conducted and the largest of the 3 values that varied less than 5% was defined as the maximum. For lung function [32] and respiratory muscle strength [33, 34], percent predicted values were calculated.

### Breath-hold duration

Maximal voluntary breath-hold duration trials were performed at total lung capacity (TLC), in a sitting position and with nose clip in place. To prevent hyperventilation prior to breath-holding measurements, the experimenter controlled the subjects’ breathing patterns visually. Subjects were instructed to continue breath-holding and to withstand any unpleasant feelings as long as possible. Maneuvers were continued until breath-hold duration did not increase any further, with a minimum of 3 trials per participant, always performed with 2-min breaks in-between. The largest breath-hold duration was used for further analysis.

### Hypercapnic ventilatory response

The assessment of HCVR was performed according to the rebreathing method of Read [35]. The rebreathing circuit consisted of a mouthpiece connected to the ergospirometric device (Oxycon Pro, Jaeger, Höchberg, Germany; for calibration see incremental exercise test) and a 3-way valve which allowed to switch, at the end of a normal expiration, from breathing room air (for 5 min) to breathing in and out of the spirometer (starting volume: FVC+1L), containing an initial gas mixture of 7% CO_2_ and 93% O_2_. The test was continued until end-tidal CO_2_ partial pressure (P_ET_CO_2_) reached 65 mmHg or the subjects stopped themselves.

Minute pulmonary ventilation (V̇_E_), f_R_, tidal volume (V_T_) and pressure of end-tidal CO_2_ (P_ET_CO_2_) were recorded breath-by-breath. Oxygen saturation (SpO_2_) and heart rate (HR) were monitored continuously on the forehead by means of a pulse oximeter (OxiMaxTM N-600xTM, Nellcor, Boulder, USA). The slope of the HCVR was calculated using a linear regression model, as described by Lorinc [36].

### Incremental exercise test

ICT was performed on an electromagnetically braked bicycle ergometer (Ergoline 800, Bitz, Germany) and the subjects were connected via mouthpiece and turbine to the Oxycon Pro, which was calibrated before each test.

ICT started with a 5 min resting period, during which subjects were sitting quietly on the bicycle. Thereafter, subjects were instructed to pedal with their self-selected pedaling rate of 60–80 rpm, starting at an initial workload of 60 W. The workload was then increased by 30 W every 2 min until exhaustion or until the subject could not keep the pedaling rate within ± 3 rpm of their preferred cadence. The experimenter did not encourage the subjects at any time.

Breath-by-breath pulmonary gas-exchange data, V̇_E_, f_R_, V_T_ and HR were collected continuously during the test. For the determination of blood lactate concentrations, 20 μl blood samples from the earlobe were taken at rest and at the end of each work and analyzed enzymatically (Biosen 5040, EKF Diagnostic, Barleben, Germany).

In addition, the subjects had to rate their perception of breathlessness, respiratory exertion and leg exertion every 2 min, using a visual analogue scale (VAS), which was post-hoc split into values between 0 and 10. The extremes of the scale were defined as “none” up to “maximal” leading to an immediate termination of the test due to these particular sensations. To ensure that subjects could distinguish between breathlessness and respiratory exertion [37, 38], subjects’ personal experience with these sensations were discussed comprehensively in advance. Hence, breathlessness was defined as “not getting enough air for a required effort”, respiratory exertion as “sensation of work and effort of respiratory muscles” or “how difficult it is to breathe” and leg exertion as “sensation of work and effort related to leg muscles”.

V̇O_2peak_ was taken as the highest 15 s average value of oxygen consumption (V̇O_2_) attained before the subject’s volitional exhaustion in the test and W_max_ was extrapolated proportionally according to time spent at the final workload.

ICT data, i.e. ventilation, gas exchange and HR, were analyzed (at submaximal exercise) by means of 15 s averages over the first 8 min, the longest available period completed by all subjects, whereas for lactate concentration and ratings of perceived sensations, values taken at 2, 4, 6 and 8 min were averaged. The slope of the ventilatory equivalent for CO_2_ (V̇E/V̇CO_2_) was calculated from all breath-by-breath data during cycling [39].

ICT was accepted as a maximal test if at least two of the following criteria were met: respiratory exchange ratio > 1.1, HR_max_ > 90% of the age-predicted HR_max_, i.e. 220- age, ratings on the VAS > 8, maximal lactate concentration > 8 mmol·L^-1^.

### Passive exercise and constant-load exercise test

The same experimental setting as described above was used for CET, including passive leg cycling movements, using the following protocol: after 5 min of rest, the subjects passively (without active muscle contractions) followed the cycling movements for 2 min while their feet were strapped to the pedals of the ergometer and the experimenter manually turned the pedals with the previously selected pedaling rate (ICT) which subjects subsequently maintained throughout the test. After the 2 min of passive pedalling, the workload was increased to 40% W_max_ for 2 min, subsequently to 60% W_max_ for 2 min, followed by 85% W_max_ until exhaustion or until subjects could no longer maintain the self-selected pedaling rate within ± 3 rpm. Subjects received no encouragement from the experimenter at any time.

Breath-by-breath data of ventilation, gas exchange and HR were collected during the test. Blood samples from the earlobe were taken at the beginning and at the end of each 2-min interval. Subjects marked VAS according to their perception of breathlessness, respiratory exertion and leg exertion every 2 min as described above. The time to task failure was used as a measure of exercise capacity and was recorded when the pedaling rate fell more than 3 rpm below the required pedaling rate.

To enable data comparison of the 2-min passive leg movement at steady-state, a 15-s average (from 1:30 to 1:45 min) was calculated and lactate concentration at the end of the 2-min period was used for analysis. Ventilation, gas exchange and HR data of the CET were analyzed by means of 15-s averages during the first 8 min (the longest period completed by all subjects), whereas for lactate concentration and rating of sensations, values taken at 4, 6 and 8 min were averaged.

### Data analysis

Data are expressed as means ± standard deviations. Differences between groups were assessed by performing one-tailed student’s t-tests for independent samples, except for anthropometric data and lung function where a two-tailed test was applied. For resting V.E, steady state data of relaxed quiet breathing (4th and 5th min) during ICT and CET were averaged and then compared to passive cycling or exercise, using one-tailed paired t-tests. Statistical significance was accepted at p < 0.05. Statistical evaluation was processed in IBM SPSS Statistics version 19.0 (SPSS, Chicago, IL, USA).

## Results

Between the YOGA and the CON group, no differences were found with regard to age, height, body mass, body mass index (BMI) (Table 1) and recreational exercise activities (mean 2.7 ± 1.7 vs. 3.4 ± 1.0 h wk^-1^, respectively; p = 0.343).

**Table 1:** Anthropometric data of yoga and control groups.

	YOGA	YOGA	CON
N	9	6	6
Age (years)	36 ± 6	35 ± 6	31 ± 5
Height (cm)	170 ± 4	171 ± 4	169 ± 5
Body mass (kg)	58 ± 4	59 ± 2	60 ± 4
BMI (kg·m^-2^)	20 ± 1	20 ± 1	21 ± 2

Data is given as mean ± standard deviation. No significant differences were found between both yoga (YOGA) and control (CON) groups. BMI, body mass index.

### Lung function and respiratory muscle strength

Lung function and respiratory muscle strength of the YOGA and the CON group are shown in Table 2. Lung function did not differ between the 2 groups. MIP and SNIP did not differ between the 2 groups while MEP was significantly lower in the YOGA compared to the CON group.

**Table 2:** Lung function, respiratory muscle strength and breath-hold duration.

	YOGA		CON	
	absolute	% pred.	absolute	% pred.
Lung function				
FVC (l)	4.6 ± 0.6	124 ± 15	4.3 ± 0.3	115 ± 8
FEV_1_ (l)	3.6 ± 0.5	112 ± 13	3.6 ± 0.3	109 ± 9
FIV_1_ (l)	4.1 ± 0.5	113 ± 14	4.0 ± 0.3	113 ± 8
PEF (l s^−1^)	7.8 ± 0.9	109 ± 12	8.0 ± 0.6	110 ± 10
PIF (l s^−1^)	5.8 ± 0.9	na	6.5 ± 0.7	na
MVV (l min^−1^)	132.8 ± 15.8	117 ± 14	144.2 ± 6.9	125 ± 7
Respiratory muscle strength				
MIP (cm H_2_O)	100 ± 12	128 ± 17	111 ± 21	144 ± 26
MEP (cm H_2_O)	140 ± 15*	144 ± 17*	161 ± 24	167 ± 24
SNIP (cm H_2_O)	93 ± 17	107 ± 19	107 ± 19	121 ± 22
Breath-hold duration				
Time (s)	70 ± 29	na	83 ± 30	na

Data is given as mean ± standard deviation. FVC, forced vital capacity; FEV_1_, forced expiratory volume in 1s; FIV_1_, forced inspiratory volume in 1s; PEF, peak expiratory flow rate; PIF, peak inspiratory flow rate; MVV, maximal voluntary ventilation; MIP, maximal inspiratory mouth pressure; MEP, maximal expiratory mouth pressure; SNIP, sniff nasal inspiratory pressure; na, not available. Significant differences between yoga (YOGA, n = 9) and control (CON, n = 6) groups are indicated.

* p < 0.05.

### Breath-hold duration

Breath-hold duration did not differ (p = 0.205) between groups (Table 2).

### Resting ventilation

**Fig 1:**
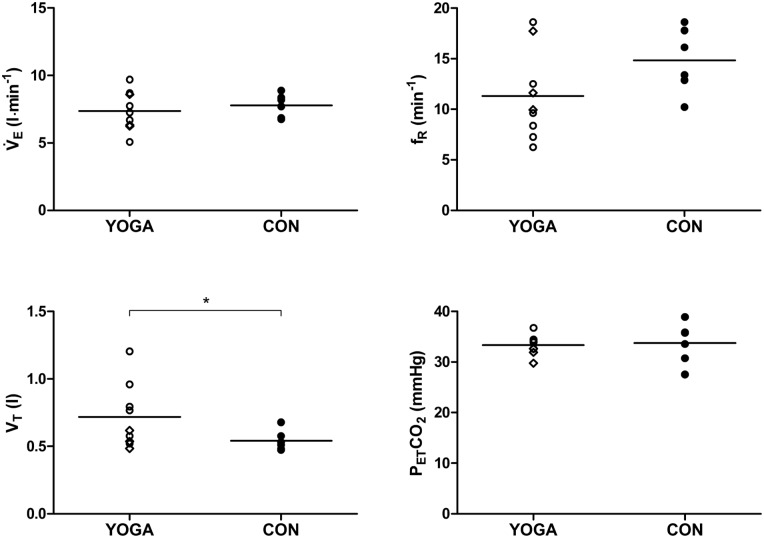
Ventilation and pattern of breathing at rest (mean ± SD) in yoga (YOGA, empty circles, empty rhombus for less fit subjects, see 3.6) and control (CON, full circles) groups. V̇_E_, ventilation; V_T_, tidal volume; f_R_, respiratory frequency; P_ET_CO_2_, end-tidal CO_2_ partial pressure. Significances are indicated for differences between YOGA and CON. * p < 0.05.

Breathing pattern differed between groups, i.e. the YOGA group showed significantly higher V_T_ (p = 0.034) and lower f_R_ (p = 0.058), although the latter exhibited great variability and did not reach statistical significance (Fig 1). V̇_E_ (p = 0.268) and P_ET_CO_2_ (p = 0.412) did not differ significantly. Resting V̇O_2_ (p = 0.443) and V̇CO_2_ (p = 0.434) did not differ between the YOGA and CON groups (data not shown).

### Hypercapnic ventilatory response

**Fig 2:**
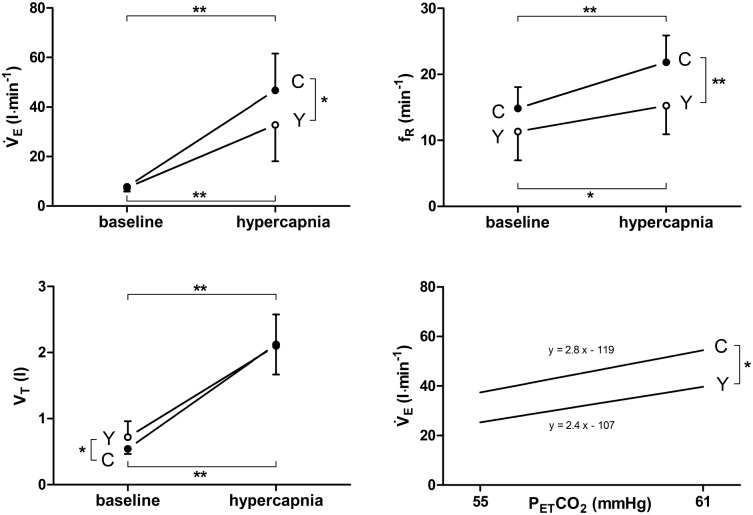
Respiratory data (mean ± SD) of yoga (Y) and control (C) groups at baseline (spontaneous breathing at rest) and during hypercapnia (achieving 55–61 mmHg end-tidal CO_2_ partial pressures [P_ET_CO_2_]). V̇E, ventilation; VT, tidal volume; fR, respiratory frequency. Bottom right: Slope of the average regression lines (y) between V̇_E_ and P_ET_CO_2_. Significances are indicated for differences between Y and C and changes from baseline to hypercapnia. * p < 0.05, ** p < 0.01.

The average ventilatory level in the range between the point where all subjects started to increase ventilation and no subject had quit the test (i.e. P_ET_CO_2_ between 55 and 61 mmHg, average P_ET_CO_2_ 58 ± 0.1 mmHg) was significantly different between the YOGA and CON groups, i.e. V̇_E_ (p = 0.048) and f_R_ (p = 0.006) were significantly lower in YOGA whereas V_T_ (p = 0.466) was similar compared to CON (Fig 2). HCVR-slopes (YOGA: 2.4 ± 0.7 vs. CON: 2.8 ± 0.8; p = 0.139) were not statistically different between the two groups.

### Passive exercise

**Fig 3:**
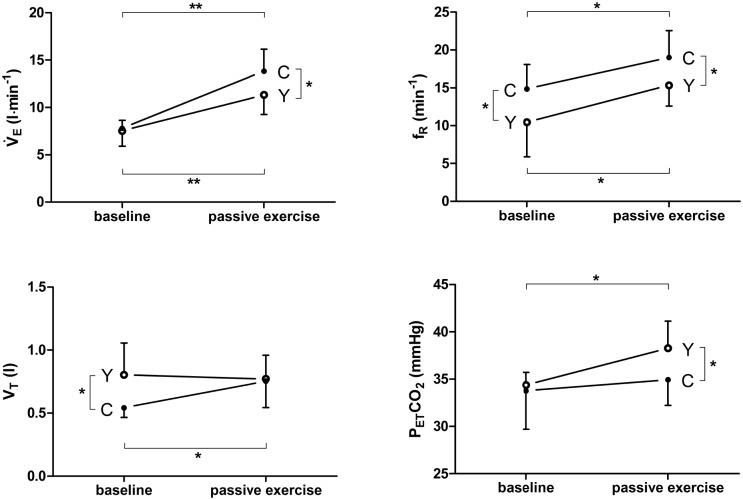
Respiratory data (mean ± SD) for the reduced dataset of yoga (Y, n = 6, see 3.6) and control (C) groups at baseline and during passive exercise. V̇_E_, ventilation; V_T_, tidal volume; f_R_, respiratory frequency; P_ET_CO_2_, end-tidal CO_2_ partial pressure. Significances are indicated for differences between Y and C and changes from baseline to passive exercise. * p < 0.05, ** p < 0.01.

Changes in the ventilatory response from baseline to passive exercise are illustrated in Fig 3. V̇_E_ increased significantly from baseline to passive exercise in both the YOGA and CON groups, with CON showing a higher V̇_E_ during passive leg movement (p = 0.039). In the YOGA group, V_T_ remained constant while f_R_ and P_ET_CO_2_ increased significantly. In the CON group, both V_T_ and f_R_ increased significantly with no change in P_ET_CO_2_. V̇O_2_ (YOGA: 250 ± 43 to 429 ± 57 ml min^-1^; p < 0.001 and CON: 239 ± 29 to 459 ± 137 ml min^-1^; p = 0.006) and V̇CO_2_ (YOGA: 226 ± 43 to 381 ± 72 ml min^-1^; p = 0.002 and CON: 218 ± 27 to 427 ± 101 ml min^-1^; p = 0.003) increased significantly from baseline to passive exercise in both groups.

### Incremental exercise test and constant load exercise test

**Fig 4:**
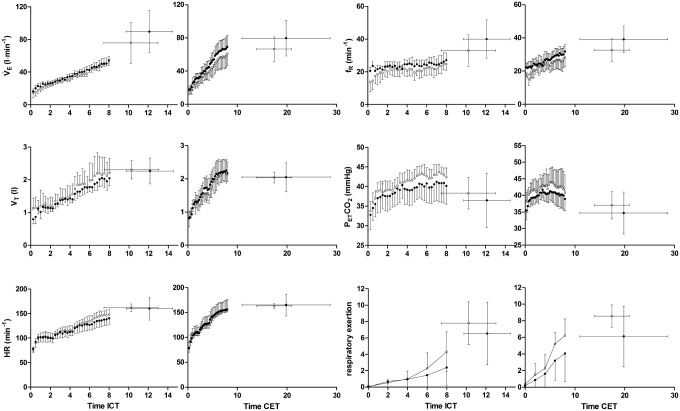
Time course of 15s data (mean ± SD) of the reduced data set (YOGA, n = 6) during the first 8 min of ICT and during the first 8 min of CET and at exhaustion of the yoga (empty circles) and control (full circles) groups. V̇_E_, ventilation; V_T_, tidal volume; f_R_, respiratory frequency; P_ET_CO_2_, end-tidal CO_2_ partial pressure; HR, heart rate.

The YOGA group showed a significantly lower W_max_ (p = 0.024) and a trend towards lower V̇O_2peak_ relative to body mass (p = 0.057). Since W_max_ is the baseline for endurance exercise testing, data of the YOGA group was analyzed using a subgroup of those 6 YOGA subjects with the highest W_max_. Anthropometric data (see Table 1) and recreational exercise activities (mean 2.8 ± 2 vs. 3.4 ± 1 h·wk^-1^; p = 0.545) of this subgroup did not differ from CON. Also, the reduced data set revealed no significant difference between YOGA and CON in W_max_, V̇O_2peak_ and endurance capacity (17.29 ± 3.30 vs. 19.56 ± 8.51 min; p = 0.276). During submaximal ICT, the YOGA subgroup showed a trend towards lower and f_R_. For the CET, no differences were found in any respiratory or metabolic parameter during the first 8 min (Table 3) or at the time of exhaustion (data not shown). Fig 4 provides a visual illustration of the time course of variables in the ICT and CET in this reduced data set.

**Table 3:** Incremental cycling and constant-load endurance cycling data of the reduced data set.

	YOGA (n = 6)	CON (n = 6)	p-Value
ICT (0–8 min)			
V̇_E_ (l·min ^-1^)	33 ± 2	36 ± 3	0.094
V_T_ (l)	1.6 ± 0.3	1.5 ± 0.1	0.193
f_R_ (min^-1^)	21 ± 4	24 ± 2	0.085
P_ET_CO_2_ (mmHg)	41 ± 4	39 ± 3	0.146
V̇O_2_ (ml·min^-1^)	1394 ± 109	1352 ± 38	0.145
V̇CO_2_ (ml·min^-1^)	1269 ± 44	1275 ± 67	0.427
V̇_E_/ V̇CO_2_	25.7 ± 2.9	29.0 ± 6.0	0.257
Lactate (mmol·l^-1^)	2.0 ± 1.1	1.8 ± 0.5	0.314
HR (beats·min^-1^)	120 ± 9	116 ± 10	0.266
Perception of breathlessness (points)	1.1 ± 0.8	0.4 ± 0.2	0.106
Respiratory exertion (points)	1.6 ± 0.7	1.1 ± 1.2	0.233
Leg exertion (points)	1.8 ± 0.7	2.0 ± 1.6	0.439
W_max_ (W)	185 ± 42	214 ± 35	0.114
V̇O_2peak_ (ml min^-1^·kg^-1^)	40 ± 6	43 ± 7	0.222
CET (0–8 min)			
V̇_E_ (l·min ^-1^)	40 ± 8	46 ± 8	0.139
V_T_ (l)	1.7 ± 0.2	1.7 ± 0.3	0.353
f_R_ (min^-1^)	24 ± 4	26 ± 3	0.112
P_ET_CO_2_ (mmHg)	42 ± 4	40 ± 2	0.144
V̇O_2_ (ml·min^-1^)	1601 ± 249	1683 ± 235	0.273
V̇CO_2_ (ml·min^-1^)	1538 ± 235	1677 ± 228	0.172
Lactate (mmol·l^-1^)	2.7 ± 0.9	3.0 ± 0.6	0.125
HR (beats·min^-1^)	130 ± 5	130 ± 15	0.469
Perception of breathlessness (points)	2.1 ± 1.6	1.3 ± 1.1	0.194
Respiratory exertion (points)	3.1 ± 1.2	2.0 ± 1.7	0.105
Leg exertion (points)	3.5 ± 1.5	3.4 ± 1.3	0.431

Data is given as mean ± standard deviation at submaximal level during the first 8 min of the incremental cycling test (ICT) and during the first 8 min of constant-load exercise test (CET) of the reduced data set. V̇_E_, ventilation; V_T_, tidal volume; f_R_, respiratory frequency; P_ET_CO_2_, end-tidal CO_2_ partial pressure; V̇O_2_, oxygen consumption; V̇CO_2_, carbon dioxide production; Lactate, blood lactate concentration; HR, heart rate; W_max_, maximal workload; V̇O_2peak_, maximal oxygen consumption. No significant differences were found between yoga (YOGA) and control (CON) groups

## Discussion

The major findings of this study were that, despite of differences in breathing pattern and respiratory regulation, YOGA participants did not show enhanced breath-hold duration nor increased exercise capacity, in contrast to our initial hypothesis. YOGA subjects, compared to CON, showed a permanent change in their breathing pattern at rest, characterized by larger V_T_. Also YOGA subjects showed a blunted ventilatory response to chemoreceptor and mechanical stimulation, thus supporting the view that long-term performance of breathing exercises during yoga practice affects spontaneous respiratory regulation. However, even when participants were matched for their maximum exercise capacity (W_max_), these differences did not translate into improved endurance capacity during the constant load exercise test.

### Effect of yoga on resting breathing pattern

In YOGA subjects, spontaneous, brainstem-driven breathing pattern at rest was altered in that V_T_ was significantly increased, in accordance to previous investigations [3, 9, 10], even though differences in f_R_ did not reach statistical significance. It appears that regular practice of volitional controlled breathing affects the brainstem respiratory center, leading to a permanent modification of the non-volitional, spontaneous breathing regulation, e.g. breathing pattern in YOGA. In spite of differences in breathing pattern at rest, however, there was no detectable between-group difference in V̇_E_, V̇_E_/V̇O_2_ (data not shown) and P_ET_CO_2_, indicating that the change in respiratory regulation specifically affected breathing pattern rather than overall ventilation at rest.

### Effect of yoga on pulmonary function and respiratory muscle strength

Although in the present study yoga experts were investigated, their lung function did not differ from CON, similar to findings in some of the previous studies [3, 13, 14] but in contrast to several others [2, 8–12]. Likewise, a difference in respiratory muscle strength was expected as result of increased neuromuscular coordination in the YOGA group due to regular practice of voluntary activation of respiratory muscles during yoga breathing, but this was also not the case. Instead, MEP was in fact even smaller in the YOGA group.

The reasons for this controversy in the literature regarding lung function and respiratory muscle strength are not immediately clear. Possibly respiratory exercises during yoga practice do not sufficiently enhance neuromuscular coordination or the contractions of respiratory muscles are not intense enough to improve muscle strength. In this light, the different results found by studies using healthy young individuals might simply reflect the low impact of yoga in these parameters.

### Effect of yoga on stimulated ventilation

Although HCVR slopes in this study did not differ, contrasting with previous studies [13, 15], the two groups had significant different V̇_E_ at similar P_ET_CO_2_. Together with similar V̇_E_ at rest, this clearly indicates a difference in chemosensitivity to hypercapnia. In this context it is necessary to point out that previous investigators [13, 15] did not clearly indicate the evaluation method of HCVR, which might be a critical point, when comparing results of different studies. A change in chemosensitivity seems plausible as yogic subjects are repetitively exposed to hypercapnia during specific respiratory exercises, as reported by Miyamura et al. (2002) [6]. It is noteworthy, however, that in spite of the seemingly reduced chemosensitivity to hypercapnia YOGA did not display increased breath-hold time compared to CON. The reason may be based in the fact that breath-hold time is not only limited by the increased CO_2_-drive to breathe but also by the complete and extended suppression of pulmonary stretch receptor activity [40–42], while with pranayama techniques ventilation is highly controlled but in most cases breathing is not fully stopped.

In the present study, f_R_ was substantially lower in YOGA during the hypercapnic condition, while V_T_ was similar and therefore the difference in V̇_E_ mainly resulted from lower f_R_. This might have contributed, at least in part, to the reduced ventilation of YOGA in the HCVR as slow f_R_ itself was shown to substantially decrease HVR and HCVR in men [43]. However, Spicuzza et al. [15] also showed that a change in chemosensitivity was not only a function of a change in f_R_, i.e. yogic subjects did not increase ventilation to values similar to controls when increasing f_R_ volitionally.

The current passive leg movement trial was designed to compare ventilatory responses resulting from afferent mechanoreceptor activity without the influence of efferent, i.e. corollary discharge (central command) to the respiratory centre with motor output to leg muscles. During passive movement, YOGA and CON increased V̇_E_ over resting values, but YOGA showed a reduced ventilatory response to passive leg movement compared to CON. This supports the observation of reduced chemosensitivity in YOGA, as YOGA subjects tolerated a higher P_ET_CO_2_ than CON also during passive leg movement. The reduced ventilatory response further indicates that long-term performance of breathing exercises affects spontaneous respiratory regulation when the respiratory drive is increased by afferent activity not typical for yoga, e.g. mechanoreceptor stimulation.

### Effect of yoga on exercise performance

Interestingly, YOGA showed a smaller W_max_ than CON in spite of similar amounts of habitual physical activity. Even in the YOGA sub-group of similar W_max_ to CON, the suggested positive effects of yoga on endurance performance could not be demonstrated, contrary to our hypothesis based on previous literature [1, 14, 20–25]. While ventilation and breathing frequency showed a trend towards lower values in YOGA during submaximal ICT, V̇_E_/V̇CO_2_ slopes, that can be seen as an indicator as ventilatory chemosensitivity [44] in spite of the possible influence of cycling efficiency [45] and the proportion of dead-space to tidal volume [46] in this variable, did not differ between groups. Also, during submaximal CET changes in ventilation and gas exchange were similar between YOGA and CON. Thus, it is not surprising that exercise endurance capacity was also similar between groups, as the hypothesized reduced ventilatory drive was absent. Finally, although subjective perception of respiratory sensations did not differ significantly between the two groups, perception of breathlessness and of respiratory exertion was actually slightly increased in YOGA. One possible explanation for this is that YOGA subjects are more susceptible to perceive breathlessness and respiratory exertion due to regular performance of breathing exercises, including a specific focus on perception of breathing.

Taken together, our findings suggest that yoga does not produce an exercise training stimulus large enough to induce measureable training responses during whole-body exercise in young, healthy individuals, as demonstrated by similar HR in both groups at any given absolute exercise intensity. Indeed, one session of YOGA at beginner-level was shown to have an equivalent intensity to treadmill walking at 3.2 km.h^-1^ [47], an intensity which is unlikely to improve exercise capacity in young, healthy individuals. From a longitudinal perspective, studies that have reported increased V̇O_2max_ with yoga training were performed mostly with sedentary elderly [1] or untrained subjects [14], i.e. subjects whose V̇O_2max_ is more likely to improve, while subjects of the present study normally performed 2.7 ± 1.7 h of exercise training per week. In fact, the beneficial effects of yoga on exercise performance in populations such as chronic heart failure patients might be potentiated due to the exacerbated dyspnea that these patients normally exhibit during exercise, as a result of excessive sympathetic stimuli [48]. Regardless of the mechanism, however, yoga or simply practicing respiratory exercises seem to be of benefit for different patient populations, and thus should not be disregarded as a therapeutic option for specific groups based on the present findings [48].

### Technical considerations and limitations

This study was performed on female participants, who possess different chemosensitivity and respiratory control compared with male individuals, even when body mass differences are taken into account [49], which is most likely related to sexual hormone concentrations [50, 51]. In spite of these differences, men and women seem to benefit from respiratory muscle training to a similar extent [52], and also present similar responses of diaphragmatic fatigue following exercise [53] and similar respiratory responses to unloading of the respiratory muscles [54]. Thus, it is tempting to speculate that male yogic individuals would show a similar response with regards to exercise performance compared to our female participants, although this hypothesis requires further testing.

Even though the present studies suggest that yoga practice does not give healthy individuals an advantage during submaximal whole-body exercise, longitudinal studies are required to confirm this hypothesis. Fatigue of the respiratory muscles is just one of many factors influencing whole-body exercise performance, and even though our participants were matched for physical activity level (excluding the hours spent practicing yoga), age and body weight, we cannot exclude the possibility that performance during CET was not improved due to a non-controlled factor, overcoming any possible advantages of the reduced ventilatory response of yogic individuals. In the specific case of female participants, even unexpected differences between the two groups in iron levels—related or not to dietary differences—or haemoglobin concentration, which impact oxygen carrying capacity during exercise, could have affected performance [55]. In this context, it is important to keep in mind that the sub-group of yoga participants was also matched to our control individuals for peak exercise capacity and V̇O_2peak_, which is especially sensitive to differences in factors such as training status or O_2_ carrying capacity, strengthening this comparison. Although one might suspect that subjects performing YOGA could favor vegetarianism, nutritional reports of our subjects show that the number of subjects not being vegetarian was similar in both groups and that all participants included several iron-rich foods in their daily meals. Thus we are confident that the nutritional status and the potential effect on hemoglobin did not systematically affect the outcome of our study.

Finally, the sample size was relatively small, and thus more prone to type II errors. However, our post hoc analyses showed that power for the measurements of breathing pattern at rest and ventilatory response to passive and active exercise ranged from 55–78%, in addition to being in line with what is commonly reported in the literature for yogic individuals (that is, decreased ventilatory response to different stimuli compared to non-yogic individuals). We did not, however, test our participants for HVR or ventilatory responses to stimulation of metaborreceptors, which would have expanded our comparison and given a more complete picture of the ventilatory response of yogic individuals to the different stimuli present during exercise. Nonetheless, when comparing exercise capacity during CET, our results, if anything, suggest a decreased endurance capacity for yogic individuals, which were already matched to peak exercise capacity during an exercise test, in opposition to any beneficial effect that a blunted ventilatory response to exercise could have on performance.

## Conclusion

This study showed that long-term yoga practice can alter the automatic output of the brainstem respiratory center in resting conditions and also reduce hypercapnic respiratory drive, possibly as the result of repetitive exposure to hypercapnia during specific respiratory exercises. However, these changes do not seem to give healthy young yogic individuals an advantage during whole-body exercise.
